# Synergistic effects of Buyang Huanwu decoction and embryonic neural stem cell transplantation on the recovery of neurological function in a rat model of spinal cord injury

**DOI:** 10.3892/etm.2015.2248

**Published:** 2015-02-02

**Authors:** MIN ZHANG, YONG CHAI, TONGSHEN LIU, NING XU, CHENG YANG

**Affiliations:** 1 Department of Anatomy, Binzhou Medical University, Yantai, Shandong 264003, P.R. China; 2 Morphology Laboratory, Binzhou Medical University, Yantai, Shandong 264003, P.R. China; 3 Department of Oncology, Affiliated Hospital of Binzhou Medical University, Binzhou, Shandong 256603, P.R. China

**Keywords:** traditional Chinese medicine, Buyang Huanwu decoction, neural stem cells, spinal cord injury, transplantation

## Abstract

The aim of the present study was to investigate the therapeutic effect of a combined treatment of Buyang Huanwu decoction (BYHWD), a well-known formula of traditional Chinese medicine, and neural stem cells (NSCs) on spinal cord injury (SCI) and the associated underlying mechanisms. A SCI model was established by surgery via a complete transection of the T10 vertebra of female Sprague-Dawley rats. Gelatin sponges were used to absorb NSCs labeled with the thymidine analog, 5-bromo-2-deoxyuridine (BrdU), and were transferred into the transected spinal cords. BYHWD was administered once a day by introgastric infusion. Motor functions of the hind limbs were evaluated using the 21-point locomotor rating scale developed by Basso, Beattie and Bresnahan (BBB). The fate of the transplanted NSCs under the various conditions was examined by double immunofluorescence staining, using markers for neurons, astrocytes and oligodendrocytes, with BrdU. Ultrastructural changes of the SCI site following the various treatments were examined under a transmission electron microscope. The number of double positive cells for glial fibrillary acidic protein and BrdU in the BYHWD + NSC group was significantly decreased when compared with that in the NSC group (P<0.05). However, the number of cells that were labeled double positive for myelin basic protein and BrdU, as well as neuron specific enolase and BrdU, was greater in the BYHWD + NSC group when compared with the NSC group. Electron microscopy demonstrated that treatment with BYHWD combined with NSCs significantly alleviated demyelination. Results from the BBB motor function test exhibited a significant improvement in the BYHWD + NSC group when compared with the SCI, BYHWD and NSC only groups. In conclusion, the results demonstrated that the traditional Chinese medicine formula, BYHWD, exerted an effect on the differentiation and migration of NSCs. Combining the administration of BYHWD with NSCs was shown to have a synergistic effect on the recovery of neurological function, mitigating the progress of demyelination or ameliorating the recovery of myelination.

## Introduction

Neurons in the central nervous system (CNS) of adult mammals were not considered to be able to undergo repair or regeneration (1). Since neural stem cells (NSCs) were first isolated from the adult mammalian CNS, NSCs have become increasingly studied due to their unique self-renewal capacity and multiple differentiation potential (2,3). Characteristics of NSCs include the generation of new cells and the creation of an environment that is good for axonal regeneration; thus, NSCs have become beneficial to the repair of spinal cord injury (SCI) (4–7). However, their potential is very limited in adult models. Firstly, the adult CNS can only provide a small number of new NSCs. In addition, the environment of the injured CNS is detrimental to the survival and differentiation of NSCs (8–10). To combat these two disadvantages, the repair strategy should be adjusted to increase the number of NSCs through transplantation and alter the local environment, respectively (11,12).

To date, the embryonic CNS is considered to be the main source of NSCs. NSCs derived from the embryonic neural tube present a unique advantage, as they have the same origin as the cells of the SCI lesion (13–15). Increasing the number of NSCs can be achieved via the expansion of NSCs *in vitro*, of which there are two methods that are currently used: Neurosphere culture (2) and adherent culture (16).

Buyang Huanwu decoction (BYHWD), recorded in ‘Yilin Gaicuo’ (Correction on Errors in Medical Classics), has been used for the treatment of stroke-induced disability and for improving neurological functional recovery in China for hundreds of years (17). In previous studies, BYHWD has been widely applied for conditions of the nervous system, and has been shown to exert neuroprotective effects on cerebral ischemia-reperfusion following injury (18–21), promoting peripheral nerve regeneration *in vivo* (22), enhancing functional recovery following spinal cord injury (23) and stimulating the growth and differentiation of neural progenitor cells *in vitro* (24). However, the effects of BYHWD on the differentiation of transplanted NSCs in an injured spinal cord, as well as the associated mechanisms underlying the improvements to the SCI symptoms, have not yet been reported.

In the present study, the effects of this formula on the differentiation of transplanted NSCs were investigated, with the aim to observe the synergistical effect on the recovery of neurological function between NSCs and BYHWD in the treatment of SCI in rats. In addition, the present study investigated the possible mechanisms of acquiring a higher BBB score following combined administration of BYHWD and NSCs in a model of SCI.

## Materials and methods

### Preparation of embryonic neural tube-derived NSCs

Embryonic neural tube-derived NSCs were cultured according to previously outlined methods (25). The posterior segment of the neural tubes from an embryonic Sprague-Dawley (SD) rat (11.5 days) were collected. The morning of the day when a vaginal plug was found was defined as embryonic day 0.5 *post coitum*. The neural tubes were cut into sections and dissociated mechanically under an anatomical microscope (SZ2-ILST; Olympus Corporation, Tokyo, Japan), under sterile conditions in the tissue culture hood. Subsequently, the samples were transferred to a centrifuge tube that contained 1 ml Hanks balanced salt solution (HBSS; 14185-052; Invitrogen Life Technologies, Carlsbad, CA, USA). Following filtration with a 200-mesh sieve, the samples were centrifuged at 300 × g for 5 min and resuspended with Dulbecco’s modified Eagle’s medium: Nutrient Mixture F/12 (DMEM/F12; 1:1; SH30126.FS, Hyclone, Shanghai HuiYing Biotechnology Co. Ltd., Shanghai, China) containing 2% B27 (17504-044; Invitrogen Life Technologies) and 20 ng/ml basic fibroblast growth factor (bFGF; PMG0035; Invitrogen Life Technologies). The cell suspension was adjusted to a concentration of 1×10^6^ cells/ml, plated and incubated at 37°C in a 5% CO_2_ incubator. Following culture for 3–4 days, half of the medium was replaced. After 7–9 days, the cells were passaged. The second passage of the NSCs were collected, and the formed neurospheres were dispersed into a single cell suspension and transferred to a culture flask. Next, 5-bromo-2-deoxyuridine (BrdU) was added to the culture flask at a final concentration of 10 μM (26). The newly formed neurospheres were dispersed into a single cell suspension after BrdU labeling for 48 h. The cell suspension was centrifuged at 300 × g for 5 min, washed with HBSS, centrifuged at 300 × g for 5 min and resuspended in complete DMEM/F12 culture media. The cell concentration was adjusted to a final density of 5×10^9^ cells/ml prior to transplantation.

### Composition and preparation of BYHWD

BYHWD consists of the following ingredients: Radix Astragali (120 g), also known as huáng qí, is the root of *Astragalus membranaceus* (Fisch.) Bge. var. *mongholicus* (Bge.) Hsiao; Radix Angelicae Sinensis (6 g), also known as dang gui, is the root of *Angelica sinensis* (Oliv.) Diels; Radix Paeoniae Rubra (4.5 g), also known as chi shao, is the root of *Paeonia lactiflora* Pall.; Rhizoma Chuanxiong (3 g), also known as chuan xiong, is the root of *Ligusticum chuanxiong* Hort.; Semen Persicae (3 g), also known as tao ren, is the dry ripe seed of *Prunus persica* (L.) Batsch; Flos Carthami (3 g), also known as hong hua, is the flower of *Carthamus tinctorius* L.; and Lumbricus (3 g), also known as di long and *Pheretima aspergillum* (perrier). All the herbs were orthodox drugs that were purchased from the Traditional Chinese Medicine Association of the Affiliated Hospital of Binzhou Medical University (Binzhou, China), and had been extracted according to standard methods outlined in the Chinese Pharmacopoeia (27). The mixture was decocted to yield a final concentration of 2 g crude drug/ml, which was stored at 4°C for further use (28).

### Animal grouping, SCI model establishment and drug administration

In total,78 female SD rats (weight, 220–250 g) were purchased from the Experimental Animal Center at Shandong Green Leaf Pharmaceutical Co., Ltd. (Yantai, China). The rats were randomly divided into five groups, which included the sham-operated control (sham control; 6 rats), SCI (18 rats); BYHWD (18 rats), NSC transplantation (18 rats) and NSCs transplantation combined with BYHWD (BYHWD + NSCs; 18 rats) groups. Three time points were selected, 7, 14 and 28 days, where each group of rats underwent examination. Surgical procedures on the rats during the experiment were performed following the guiding principles of care (29), and were consistent with the National Institutes of Health Guide for the Care and Use of Laboratory Animals (30). All experiments involving rats in the present study were approved by the Ethics Committee for animal care and use of Binzhou Medical University (Yantai, China). Anesthesia was achieved by an intraperitoneal injection of 4% chloral hydrate (0.1 ml/kg). A laminectomy was performed for the rats in the sham control group. The spinal cords of the rats in the remaining four groups underwent complete transection at the T10 vertebra. During surgery, gelatin sponges (2×2×2 mm^3^) containing 10 μl BrdU-labeled NSCs (5×10^9^ cells/ml) were transplanted into the SCI site in the NSCs and BYHWD + NSCs groups. In the SCI control group, gelatin sponges containing 10 μl DMEM/F12 complete culture media were transplanted into the SCI site.

The dosage of BYHWD was selected according to the conversion factor of the dosage between humans and rats: Rat dose (mg/kg) = W × human dose (mg/kg), where W is the conversion factor (6.25). The BYHWD dose selected was ~14.8 g/kg/day. Thus, the rats in the BYHWD and BYHWD + NSCs groups were administered BYHWD at this dose by introgastric infusion once a day, while the same volume of saline was administered to the rats in the NSC group. Postoperative care was conducted according to the National Institutes of Health guidelines (31).

### Behavioral analysis

All the rats were evaluated by two trained examiners via a double blind method. The Basso, Beattie and Bresnahan (BBB) locomotor rating scale was used to determine motor function prior to the injury and transplantation, at 24 h after the surgery, and then weekly for four weeks. The open field locomotor activity score was determined by observing and calculating the behaviors involving the movement of all joints of the hindlimbs, plantar placement, forelimb and hindlimb coordination, the trunk stability and tail position, according to the BBB protocol. Each session lasted for 4 min. Scores from the two examiners were averaged for each rat, and scores ranged between 0 and 21 (0, no movement; 21, normal movement) (32).

### Tissue processing

Rats were deeply anesthetized by an intraperitoneal injection of 4% chloral hydrate. Subsequently, 0.9% saline solution (37°C; 200 ml) was perfused through the heart, followed by 4% paraformaldehyde in 0.1 M phosphate buffer (pH 7.4) at 4°C (200 ml). The spinal cords were dissected, post-fixed with 4% paraformaldehyde at 4°C for 2 h, and subjected to graded dehydration, xylene transparent clearing, wax infiltration, paraffin embedding and sectioning.

### Immunofluorescence staining

Primary antibodies were used as follows: Mouse anti-BrdU (monoclonal IgG; 1:100; ZM-0013; Beijing Zhongshan Golden Bridge Biotechnology Co., Ltd., Beijing, China), rabbit polyclonal anti-nestin (1:100; Beijing Zhongshan Golden Bridge), rabbit polyclonal anti-neuron specific enolase (NSE; 1:200; BA0535; Wuhan Boster Biological Engineering Co., Ltd., Wuhan, China), rabbit polyclonal anti-myelin basic protein (MBP; 1:150; BA0094; Wuhan Boster Biological Engineering Co., Ltd.) and rabbit polyclonal anti-glial fibrillary acidic protein (GFAP; 1:200; BA0056; Wuhan Boster Biological Engineering Co., Ltd.). Cy3-conjugated goat anti-rabbit IgG (1:100; BA1032; Wuhan Boster Biological Engineering Co., Ltd) and fluorescein isothiocyanate-conjugated goat anti-mouse IgG (1:200; ZF-0312; Beijing Zhongshan Golden Bridge, Beijing, China) were used as secondary antibodies. Paraffin-embedded sections were deparaffinized, rehydrated and washed three times with 0.01 M phosphate-buffered saline (PBS). The samples were subsequently incubated with 0.1 M HCl containing 0.4% pepsin (Sigma-Aldrich, St. Louis, MO, USA) for 10 min at 37°C, and then 0.2 M HCl for 30 min at 37°C. After washing three times with 0.01 M PBS, the samples were incubated with 0.01 M PBS containing 0.05% Triton X-100 for 15 min at room temperature, washed three times with 0.01 M PBS and incubated with goat serum working solution for 30 min at 37°C (33). Incubations with the appropriate primary antibody were performed overnight at 4°C. Following repeated washing with 0.01 M PBS, the sections were incubated with their respective secondary antibodies for 30 min at 37°C. Finally, the sections were washed with 0.01 M PBS, mounted with a coverslip and examined under a confocal microscope (Leica TCS SPE; Leica Microsystems GmbH, Wetzlar, Germany).

### Cell counting

For quantitative analysis of the transplanted NSCs in the spinal cord, beginning from the site of transplantation, the spinal cord of the rats was sectioned continuously at a 5-μm thickness in the transverse plane towards the rostral and caudal end. Every fifth slice was collected, resulting in six sections in each direction being obtained. GFAP and BrdU double positive cells within the white matter and gray matter of every section were counted at the same magnification (x40; Leica TCS SPE; Leica Microsystems GmbH). Area analysis of the GFAP-positive astrocytes, NSE-positive neurons and MBP-positive oligodendrocytes was assessed using Image-Pro Plus 6.0 pathological image analysis software (Media Cybernetics, Inc., Rockville, MD, USA).

### Statistical analysis

Data corresponding to the BBB scores, the number of GFAP/BrdU double positive cells and the area analysis of astrocytes, neurons and oligodendrocytes are expressed as the mean ± standard deviation. A two-way classification analysis of variance of the factorial experiment, followed by the Student-Newman-Keuls test, was performed for statistical analysis. P<0.05 was considered to indicate a statistically significant difference. Experimental data were analyzed using the SPSS 13.0 software package (SPSS, Inc., Chicago, IL, USA) to estimate the significance of the difference between changes in the data.

## Results

### NSC culture and characterization

**Figure 1:**
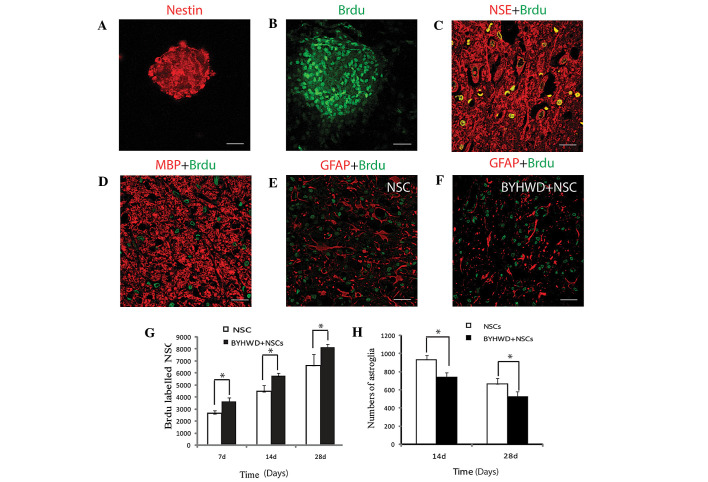
Characterization and differentiation of the cultured isolated NSCs from the rat embryo neural tube. (A) Cultured NSCs grew into neurospheres in the culture dish, expressing the neural stem cell marker, nestin. (B) NSCs continued to proliferate in the *in vitro* culture, as shown by the incorporation of BrdU (green). (C) Transplanted NSCs in the injured spinal cord differentiated into neurons, labeled with the neuron marker, NSE (red) and BrdU (green). (D) Transplanted NSCs differentiated into oligodendrocytes, expressing MBP and BrdU, and incorporated into neural networks. (E and F) BYHWD treatment exerted a suppressive effect on astrogliosis in the spinal cord injury (SCI) site, as compared with NSC treatment alone. (G) BYHWD treatment was shown to promote NSC survival at the SCI site at the three examined time points (^*^P<0.05; n=36). (H) BYHWD treatment was shown to inhibit astrogliogenesis in the surrounding SCI site at the two examined time points (^*^P<0.05; n=36). BYHWD, Buyang Huanwu decoction; NSC, neural stem cells; BrdU, 5-bromo-2-deoxyuridine; GFAP, glial fibrillary acidic protein; MBP, myelin basic protein; NSE, neuron specific enolase. Scale bars, 50 μm (A and C), 30 μm (B) and 100 μm (D, E and F).

NSCs obtained from the embryonic neural tube at 11.5 days *post coitum* were cultured in DMEM/F12 medium containing B27 and bFGF. At day 2 of *in vitro* culture, the NSCs proliferated and formed neurospheres. Between days 7 and 9, the NSCs became confluent and underwent cell passage. These undifferentiated neurospheres expressed the primitive neurofilament, nestin, which was used as a marker of NSCs (Fig. 1A).

To trace the NSCs and their fate following transplantation adjacent to the injured spinal cord site, as well as the response to the treatment of BYHWD, BrdU was supplemented into the culture media. Immunocytochemistry revealed that the BrdU was efficiently incorporated into the proliferating NSCs (Fig. 1B)

### Survival and proliferation of NSCs in the transected spinal cord

BYHWD was shown to promote the survival and proliferation of NSCs in the SCI site. Following transplantation, the grafted NSCs were easily identified primarily around the lesion site of the host spinal cord, and had integrated well with the host tissue in the NSCs only and BYHWD + NSCs groups. The total counted number of BrdU-labeled NSCs in the NSCs only transplanted group at day 7 was 2,627±259.435, while at day 14, the number was 4,460.33±508.723 and at day 28, the total number was 6,631.83±909.948. The number of BrdU-labeled NSCs in the NSC and BYHWD + NSC groups at day 28 was significantly increased when compared with the numbers at days 7 and 14; however, the difference between the numbers at days 7 and 14 were not statistically significant. In the BYHWD + NSCs group, the total counted number of BrdU-labeled NSCs at days 7, 14 and 28 were 3,643.67±281.378, 5,755.50±242.561 and 8,143.50±267.644, respectively. The number of BrdU-labeled NSCs significantly increased with time when comparing the numbers at days 7, 14 and 28. In addition, the number of BrdU-positive cells at each of the three time points in the BYHWD + NSCs group was greater compared with the number at the corresponding time point in the NSCs group (n=18; P<0.05). The number of BrdU-positive cells at day 28 was the highest among the three time points in the BYHWD + NSCs group (n=18; P<0.05; Fig. 1G).

In the control experiment, a gelatin sponge injected with 10 μl culture medium was transplanted into the SCI site. There were no BrdU-positive cells observed in the sham-operated control, SCI and BYHWD groups (data not shown).

### NSC differentiation and migration into the SCI site

To determine the fate of the transplanted NSCs and their differentiation status, immunohistochemical staining of the neuron marker, NSE, was performed. NSE/BrdU double labeling revealed that the majority of the transplanted NSCs had differentiated into neurons (Fig. 1C); however, there was little differentiation into astroglial cells (Fig. 1E and F). To assess whether the NSCs had differentiated into oligodendrocytes and were able to express MBP to protectively wrap the axons for electrical signal conduction, immunohistochemical staining of MBP was performed. The results revealed that the BrdU-labeled NSCs were closely associated with the myelin-wrapped axons (Fig. 1D).

The effect of BYHWD on NSC differentiation into neurons was analyzed at three time points (days 7, 14 and 28). The number of NSE/BrdU double labeled neurons in the three corresponding time points did not exhibit a statistically significant difference between the NSC and BYHWD + NSC groups (data not shown).

To examine the effect of BYHWD on NSC differentiation into astroglia, or the inhibition of gliosis in the SCI site, GFAP/BrdU double labeling immunofluorescence was performed. The results revealed few double labeled cells were observed in the SCI site (Fig. 1E and F). The number of GFAP-labeled cells in the BYHWD-treated group was significantly decreased when compared with the number in the NSC transplantation only group (Fig. 1H). The average optical density of GFAP immunofluorescence in the NSC only and BYHWD + NSC groups was significantly lower when compared with the BYHWD group (data not shown).

Since oligodendrocytes are the predominant myelinating cell type in the CNS, whether the transplanted NSCs were able to differentiate into oligodendrocytes in the SCI site and integrate into the nerve fiber network was investigated. MBP/BrdU double labeling immunofluorescence revealed that the BrdU-labeled cells were closely incorporated inside the network of MBP-positive nerve fibers (Fig. 1D).

### NSC migration

**Figure 2:**
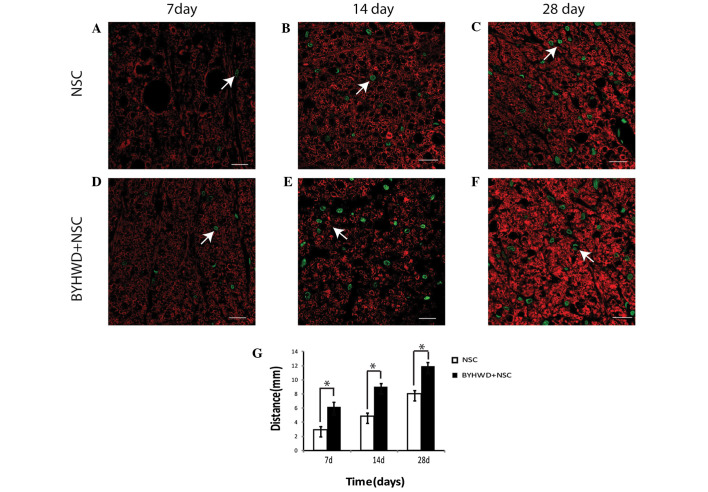
(A-F) BYHWD promotes NSC migration in the spinal cord injury site in rostral and caudal directions. NSCs were shown to migrate into the white matter and gray matter, closely integrating with the nerve tract and neural networks. At the same level of the spinal cord, there were more fluorescein immunofluorescence-labelled Brdu incorporated NSCs (white arrows) in the BYHWD-treated group (D, E and F) compared with the NSC only group (A, B and C) at the corresponding time points. (G) BYHWD promotes the migration of NSCs in the spinal cord (^*^P<0.05; n=36). BYHWD, Buyang Huanwu decoction; NSC, neural stem cell. Scale bar, 100 μm.

The distance of NSC migration into the surrounding SCI undamaged tissue is associated with improvements to the SCI symptoms. To further investigate the possible mechanisms underlying the improvements in motor behavior following NSC transplantation and treatment with BYHWD, the total distance migrated by the BrdU-labeled cells appearing in the rostral and distal sections of the SCI site was determined. In the BYHWD + NSC group, the total distance traveled at day 7 was 6.15±0.49 mm, while at day 14, the distance was 9.02±0.45 mm. By day 28, the total migration distance was 11.95±0.50 mm. In the NSC only group, at days 7, 14 and 28, the total distances traveled were 2.92±0.66, 4.85±0.46 and 8.01±0.51 mm, respectively. All the distances in the BYHWD + NSC group were significantly greater when compared with the distances in the NSC group at the respective time points (n=18; P<0.05; Fig. 2A–G).

### Ultrastructure of the SCI prior to and following the treatment

**Figure 3:**
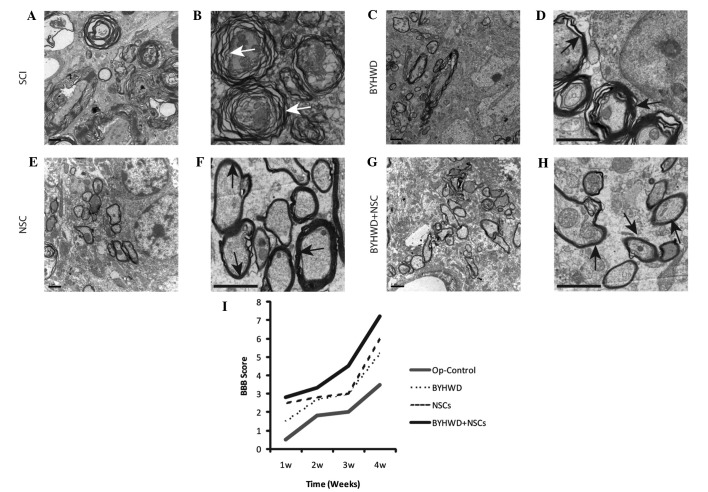
Ultrastructure of the spinal cord at the SCI site and the effect of NSCs and BYHWD treatment on SCI rehabilitation, as measured by the BBB locomotor rating scale. Ultrastructure examination revealed the effect of the different treatments on the SCI after 28 days. (A and B) No treatment resulted in onion-like demyelination (white arrow). (C and D) Treatment with BYHWD only mitigated the demyelination (black arrow). (E and F) Treatment with NSCs only further promoted the mitigation of demyelination when compared with the BYHWD and control group (black arrows). (G and H) Combination treatment with BYHWD and NSCs notably alleviated the demyelination, and the myelination wrapping the axons was shown to recover to relative normality. B, D, F and H are higher magnification compared with A, C, E and G, respectively. (I) Motor rehabilitation, as measured using the BBB locomotor rating scale. Treatment with BYHWD + NSCs demonstrated a synergistic effect on locomotion recovery. BYHWD, Buyang Huanwu decoction; NSC, neural stem cells; SCI, spinal cord injury; BBB, Basso, Beattie and Brasnahan. Scale bar, 2 μm.

Following induction of the injury, apparent changes were observed around the injury site of the spinal cord. Firstly, the myelin sheath underwent loosely onion-like demyelination, while the wrapped axons exhibited degeneration. In addition, a vacuole change was observed inside the myelinated axon (Fig. 3A and B). In the SCI models treated with BYHWD and NSC transplantation separately for four weeks, improvements were observed with regard to the tightness of the myelin sheath to varying degrees (Fig. 3C–F). However, the combined treatment of BYHWD + NSCs improved the myelination to the greatest degree when compared with the single treatments of BYHWD or NSCs (Fig. 3G and H).

### Synergy between BYHWD and NSCs leads to the recovery of neurological function

Following spinal cord surgery, the rats in the sham-operated group exhibited normal motor function, while all the rats in the four remaining groups were paralyzed, unable to stand independently and moved by pulling themselves forward with their forelimbs. Urinary incontinence was also later observed in these rats. The hindlimb locomotor activities of all the rats were evaluated using the BBB locomotor rating scale at four time points between one and four weeks after surgery. The BBB scores were shown to gradually increase over time in each group throughout the entire follow-up period, which represented gradual improvements to the hindlimb locomotion activity. In addition, BBB scores were significantly higher in the three treatment groups when compared with the SCI group in the period between two and four weeks after surgery. Compared with the BYHWD and NSCs only groups, the BYHWD + NSCs group exhibited a significantly higher score starting at the second week, while the most notable difference was observed at the fourth week following the surgery. However, the differences in the BBB scores between the BYHWD and NSCs groups were not statistically significant during the same time period (Fig. 3I).

## Discussion

In a previous study, embryonic neural tube-derived NSCs were demonstrated to have multiple potentiality to differentiate into neurons, oligodendrocytes and astrocytes *in vitro* (25). Using a pharmacological serum testing method, Sun *et al* found that BYHWD may directly promote the differentiation of neural progenitor cells (24). In the current study, the transplantation of embryonic neural tube-derived NSCs was employed in conjunction with BYHWD administration for the treatment of rats that had undergone transection of the spinal cord. The efficacy of BYHWD treatment on the survival and differentiation of the transplanted NSCs was further confirmed. The results revealed a synergy between BYHWD and NSCs in improving the neurological behavior performance, as measured using the BBB locomotor rating scale.

Previous studies have shown that NSCs exert a protective effect at the lesion site by means of multiple mechanisms, including secreting diverse neurotrophic factors and cytokines, mobilizing endogenous stem cells and replacing lost cells (11,34,35). To achieve all these protective effects, a sufficient number of surviving NSCs are required to differentiate into neural cells following transplantation. Compared with the NSCs only group, a higher number of BrdU-positive cells were observed in the NSCs + BYHWD group. Thus, BYHWD treatment was found to improve the survival rate of the NSCs. In addition, a greater area of MBP-positive oligodendrocytes was present in the rats of the NSCs + BYHWD group, which was an important indicator for remyelination at the lesion site. By contrast, a smaller number of GFAP/BrdU double positive astrocytes were present within the injured spinal cords of the NSCs + BYHWD group, indicating that BYHWD treatment suppresses injury-induced astrogliosis in the spinal cord. This may further limit scar formation or improve the reestablishment of the neuronal innervation circuit between the upper control neurons and the targets effect organs. Electron microscopy results clearly revealed that the administration of NSCs and BYHWD improved the tightness of the myelin sheath in the neural tract between the upper control neurons and the targets effect organs. However, the exact mechanisms and factors involved in the pathway of achieving the improvements, as well as the establishment of a synaptic connection, remains unclear and further investigation is required. Recent progress in self-derived induced pluripotent stem cells has enabled the prospect of patient-derived cells in the treatment of SCI and other stem cell-required treatment to become a prospective strategy (36). From the results of the present study, BYHWD was concluded to be beneficial to the survival and differentiation of NSCs into neurons and oligodendrocytes, while reducing the differentiation into astrocytes.

In the present study, rats treated with BYHWD + NSCs following SCI exhibited significantly enhanced functional recovery of the injured forelimb. Comprised of seven Chinese plant drugs, BYHWD has multiple chemical constituents. Although Yang *et al* reported that the chemical constituents of BYHWD are numerous and diverse, including flavonoids, alkaloids and saponins (37), the effective constituents of BYHWD and the mechanisms of action remain unknown. Thus, much research required to clarify the underlying mechanisms and subsequently develop this formula into a potential therapeutic reagent.

In conclusion, the results of the present study indicate that administration of BYHWD may enhance the differentiation of NSCs into neurons and oligodendrocytes, while reducing the differentiation into astrocytes, in the injured spinal cord. A synergy was identified between BYHWD and NSCs that resulted in the recovery of neurological function, which indicated that BYHWD may aid in the treatment of SCI in humans. However, further studies investigating the underlying mechanisms of BYHWD are required in the future.
